# Modeling oxidative injury response in human kidney organoids

**DOI:** 10.1186/s13287-022-02752-z

**Published:** 2022-02-21

**Authors:** Aneta Przepiorski, Thitinee Vanichapol, Eugenel B. Espiritu, Amanda E. Crunk, Emily Parasky, Michael D. McDaniels, Dave R. Emlet, Ryan Salisbury, Cassandra L. Happ, Lawrence A. Vernetti, Matthew L. MacDonald, John A. Kellum, Thomas R. Kleyman, Catherine J. Baty, Alan J. Davidson, Neil A. Hukriede

**Affiliations:** 1 Department of Developmental Biology, University of Pittsburgh, School of Medicine, 3501 5th Ave., 5061 BST3, Pittsburgh, PA 15213 USA; 2 Department of Molecular Medicine and Pathology, School of Medical Sciences, University of Auckland, Auckland, New Zealand; 3 Center for Critical Care Nephrology, University of Pittsburgh, School of Medicine, 3501 5th Ave., 5061 BST3, Pittsburgh, PA 15213 USA; 4 Department of Psychiatry, University of Pittsburgh, School of Medicine, 3501 5th Ave., 5061 BST3, Pittsburgh, PA 15213 USA; 5 Department of Computational and Systems Biology, University of Pittsburgh, School of Medicine, 3501 5th Ave., 5061 BST3, Pittsburgh, PA 15213 USA; 6 Renal-Electrolyte Division, Department of Medicine, University of Pittsburgh, School of Medicine, 3501 5th Ave., 5061 BST3, Pittsburgh, PA 15213 USA

**Keywords:** Kidney organoids, iPSCs, Renal injury, ROS, Hemin, Mitochondria, Cytochrome C

## Abstract

**Background:**

Hemolysis occurs in many injury settings and can trigger disease processes. In the kidney, extracellular hemoglobin can induce damage via several mechanisms. These include oxidative stress, mitochondrial dysfunction, and inflammation, which promote fibrosis and chronic kidney disease. Understanding the pathophysiology of these injury pathways offers opportunities to develop new therapeutic strategies.

**Methods:**

To model hemolysis-induced kidney injury, human kidney organoids were treated with hemin, an iron-containing porphyrin, that generates reactive oxygen species. In addition, we developed an induced pluripotent stem cell line expressing the biosensor, CytochromeC-GFP (CytoC-GFP), which provides a real-time readout of mitochondrial morphology, health, and early apoptotic events.

**Results:**

We found that hemin-treated kidney organoids show oxidative damage, increased expression of injury markers, impaired functionality of organic anion and cation transport and undergo fibrosis. Injury could be detected in live CytoC-GFP organoids by cytoplasmic localization of fluorescence. Finally, we show that 4-(phenylthio)butanoic acid, an HDAC inhibitor with anti-fibrotic effects in vivo, reduces hemin-induced human kidney organoid fibrosis.

**Conclusion:**

This work establishes a hemin-induced model of kidney organoid injury. This platform provides a new tool to study the injury and repair response pathways in human kidney tissue and will assist in the development of new therapeutics.

**Supplementary Information:**

The online version contains supplementary material available at 10.1186/s13287-022-02752-z.

## Background

Intravascular hemolysis occurs in many disease settings such as rhabdomyolysis, sepsis, ischemia reperfusion injury (IRI), sickle cell anemia or cardiac bypass [1, 2]. Hemolytic events can lead to acute injury events, and recurrent hemolysis can eventually lead to chronic kidney disease (CKD). Under healthy conditions, cell-free hemoglobin is present in circulation at very small amounts due to scavenging proteins such as haptoglobin or hemopexin [3]. Under pathological conditions, when hemolysis occurs, cell-free hemoglobin is released in significant amounts into the circulation [4]. The sudden release of cell-free hemoglobin, also releasing heme and iron, can overwhelm the mechanisms in place that neutralize the impact, and as a result, lead to cellular injury. Iron levels are also tightly regulated in the body and are normally supplied via absorption in the gut or recycling of senescent red blood cells. Under healthy conditions ferrous iron (Fe^2+^) is bound with heme. However, if it becomes released into the circulation, it is very quickly oxidized to Fe^3+^ and Fe^4+^. Cell-free hemoglobin and heme are highly reactive and will facilitate formation of other reactive oxygen, nitrogen, and lipid molecules. Hemin is the Fe^3+^ oxidized form of heme which under serum or protein-free conditions can be used in cell culture to study cell responses [5].

Renal injury, due to hemoglobin and heme, appears to be multifactorial including cellular membrane damage, oxidative stress imbalance, reaction with other proteins and lipids, and activation of immune responses. Due the hydrophobic nature of heme, it readily binds to cellular and intracellular membranes and proteins leading to lipid peroxidation and altered protein function [6–8]. Once heme is internalized by the cells, it is catabolized by heme oxygenase 1 (HMOX1) enzyme. This reaction is rate limiting and the breakdown of heme produces biliverdin, iron, and carbon monoxide, which are released into the cytosol [9]. Even though carbon monoxide and biliverdin have anti-inflammatory effects, release of iron can trigger Fenton reactions, which generates increases in hydroxyl radicals and oxidative stress that can overwhelm the cellular antioxidant capacity leading to injury [6, 10].

Reactive oxygen species (ROS) are generated as part of normal metabolic functions [11]. Mitochondria are the most metabolically active organelles in the cell and generate the greatest amounts of ROS, releasing most of it in the form of hydroxyl. Since cell-free heme and iron are highly reactive with hydroxyl radicals, mitochondria are vulnerable to injury. Studies conducted in rats using glycerol to cause rhabdomyolysis induced acute kidney injury (AKI), show that mitochondria readily accumulate heme within hours of injury, leading to a rapid rise in oxygen consumption and corresponding increase in ROS. This immediate effect is followed by a reduction in both oxygen consumption and transmembrane potential leading to mitochondrial dysfunction [12]. Another consequence of increased ROS is reaction of hydroxyl radicals with nitric oxide (NO) to form peroxynitrite. Peroxynitrite modifies tyrosine residues on proteins to 3-nitrotyrosine [13], which leads to altered protein and enzymatic function, lipid peroxidation, and cellular damage [14]. To study ROS-mediated injury in real-time, we developed a novel biosensor induced pluripotent stem cells (iPSC) line, using CytochromeC-GFP (CytoC-GFP) [15]. CytoC is a transmembrane protein that is localized to the inner mitochondrial membrane and is part of the electron transport chain. In healthy cells CytoC acts as an electron carrier to reduce the levels of ROS produced by the mitochondria [16]. However, rapid increases in ROS (such as found in cellular injury) results in CytoC translocating to the cytoplasm where it activates the caspase cascade in the apoptotic pathway [17].

Developing targeted renal therapeutics has resulted in no approved drugs, to date. While there are several possible reasons for this, one possibility is that results from preclinical murine models do not consistently translate to human studies [18]. One way to address this shortcoming is to utilize human kidney organoids. Many protocols have now been developed to generate kidney organoids to study kidney injury and disease pathways in vitro*.* However, few models of injury have been developed, and to date, most have focused on nephrotoxin (cisplatin, gentamicin) mediated injury [19–25]. Using a simple bioreactor-based method for generating human kidney organoids from iPSCs [26, 27], we hypothesized hemin-induced ROS-mediated injury and fibrosis could be a valuable tool for evaluating potential therapeutics. Here, we show how this model can be used to test the efficacy of promising anti-fibrotic agents such as 4-(phenylthio)butanoic acid (PTBA), which we have previously shown in mouse models can ameliorate injury and fibrosis [28–32].

In this study, we developed a human kidney organoid hemolytic injury model that can be utilized to study acute injury, mitochondrial dysfunction, and fibrosis. We chose to use hemin to induce injury, as hemin is a potent ROS inducer, a central player in renal damage, and mimics the hemolytic response seen in many diseases leading to renal injury [3]. As such, we hypothesize this model can be used to extrapolate common disease pathways and focus on therapeutic outcomes that will be widely applicable a for multi-factorial renal disease.

## Methods

### iPSC culture

All work was performed in compliance with institutional guidelines (IBC201600244), and was carried out in a Class II biosafety hood with appropriate personal protective equipment. iPSCs were maintained on 10 cm cell culture dishes coated with Geltrex (Thermo Fisher) or Cultrex (R&D) and mTeSR1 (Stemcell Technologies) medium, passaged every 3–4 days. Experiments were performed with MANZ-2-2 (female) and MANZ4-37 (male) iPSC lines [33] as stated in the figures.

### Kidney organoid assays and multi-well magnetic spinner

Kidney organoid assays and setting up of magnetic stir-plate were performed as described previously with modifications to the original Przepriorski et al. 2018 protocol [27].

### Hemin treatment

Hemin (Millipore-Sigma) 10 mM stock concentration was resuspended in 0.1 M NaOH, sterile filtered and prepared fresh for every experiment. Day 14 organoids were washed thrice with DMEM-low glucose, then transferred into protein-free medium (1:1 ratio of DMEM-low glucose and Hams F-12 Nutrient mixture, 1 × HEPES (to stabilize pH) 1% penicillin/streptomycin (Gibco), and 2.5 ug/mL Plasmocin) containing hemin in a 6-well ultra-low attachment (ULA) plate. The assay was then placed on a magnetic stir plate (2mag-USA) at 120 rpm, 25% power. Except where stated, hemin concentration was at 25 µM. Control well contained equivalent volume of 0.1 M NaOH as a vehicle control. All treatments were maintained for 48 h, and thereafter washed thrice with Stage II medium (DMEM-low glucose, 10% knock-out serum replacement, 1% penicillin/streptomycin (Gibco), 1% Glutamax (Gibco), 1% HEPES, 1% MEM non-essential amino acids, 0.5% polyvinyl alcohol, 2.5 ug/mL Plasmocin) before proceeding to compound treatment. The pH of the control and hemin-containing media was tested following 48 h incubation to exclude possibility of injury due to pH changes. The pH tested was 7.7 for control and 7.55 for hemin, within the normal range shown not to affect cellular apoptosis [34].

### Compound treatments

Day 16 kidney organoids (post hemin treatment) were treated daily with UPHD25 compound [30, 31]. Stage II medium supplemented with 0.3% DMSO (Stage II-DMSO) was prepared. Firstly, a 2 × stock solution of compound was prepared in Stage II-DMSO, and a calculated amount was added to each well to make up 1 × working solution in a total of 3 mL volume, per well of a 6-well ULA plate. The plates were maintained on the magnetic stirrer at 25% power and 120 revolutions until fixation at day 26.

### RNA extraction, cDNA synthesis and qPCR

Organoids were washed in PBS and homogenized in TRIzol (Thermo Fisher). Total RNA was extracted first using Phase Separation Reagent (Molecular Research Center) and using Qiagen RNeasy kit. cDNA was synthesized using qScript cDNA SuperMix (Quanta). qPCR was performed using the Power SYBR Green reagent (Thermo Fisher) on a QuantStudio 12 Flex Real-Time PCR machine. Gene expression was calculated using the dCt method using *HPRT1* for normalization [35]. Error bars represent standard deviation of triplicate measurements. All qPCR analyses were performed in organoids derived from three independent kidney organoid assays and representative results are shown.

### RNA-Seq and analysis

Total RNA from quadruplicate samples of control and hemin treated MANZ2-2 kidney organoids (~ 100 organoids/sample) was prepared using TRIzol and Phase Separation Reagent, purified using Ambion PureLink RNA Mini Kit with in-column RNase-Free DNase I (Qiagen) treatment. All samples contained > 1 µg total RNA. All samples sequenced had a RIN value of ≥ 9.4. Library preparation was done using TruSeq Stranded mRNA (PolyA +) kit, and sequencing on Illumina Sequencing using NextSeq500. Quality control, library preparation and sequencing were performed by Health Sciences Sequencing Core, UPMC Children’s Hospital of Pittsburgh. Reads were mapped on the human genome, GRCh38.p13 using STAR [36] and counted using featureCounts [37]. Differential expression analysis was carried out using edgeR [38] and limma [39] with a threshold of log fold change = 1 and adjusted P value of < 0.05. Gene set enrichment analysis (GSEA) was performed for the hallmark gene sets and Gene Ontology (GO) classification from the MSigDB collections using the clusterProlifer package [40–43]. The RNA sequencing (RNA-Seq) data were deposited in the National Center for Biotechnology Information’s GEO database (GSE182350).

### CytoC-GFP iPSC line development and imaging

CytoC-GFP iPSC lines were generated using AAVS1 Safe Harbor Site Targeting 2.0 Complete Kit 2.0, with an all-purpose HR donor vector (System Biosciences). The CytoC-GFP construct generated in Douglas Green’s lab (Addgene #41,182) [15] was cloned into the AAVS1-SA-puro-EF1α-MCS donor vector using Cold Fusion Cloning kit (System Biosciences). The donor vector was transfected into the MANZ2-2 cell line using Lipofectamine stem reagent according to manufacturer’s specifications (ThermoFisher). When the plated cells reached 70% confluency puromycin was added at 0.5 µg/mL to select positive cells that underwent homologous recombination and integration of the donor construct. After 10 days of daily treatment with puromycin, stable colonies positive for EF1A-CytoC-GFP emerged. Colonies which had the strongest and most homogeneous expression of CytoC-GFP in the mitochondria were manually isolated and expanded to generate individual cell lines. Three cell lines were selected for further validation and experiments. Live organoids treated with hemin were imaged on a Leica Sp8 confocal in coverglass bottom 30 mm dish (Cellvis) in protein free medium using an OKO environmental chamber at 40 × water objective 1.1 NA, 0.42 µm step, 3z.

### Live MitoTracker Red CMXRos staining

Stock concentration of 1 mM MitoTracker Red CMXRos was prepared in DMSO according to manufacturer’s instructions. CytoC-GFP iPSC lines and organoids were washed with DPBS, and then incubated for 30 min in a working solution of 500 nM MitoTracker Red CMXRos in mTeSR1 or Stage 2 medium respectively. Kidney organoids were incubated for at least 1 h before imaging. Cell were then washed in DPBS and imaged in FluoroBrite™ DMEM (Gibco) on the Zeiss LSM700 confocal microscope.

### Live dihydroethidium (DHE) staining

Stock concentration of 5 mM DHE was prepared in DMSO according to manufacturer’s instructions. CytoC-GFP iPSC organoids were washed with DPBS, and then incubated for 1 h in a working solution of 5 µM DHE in stage 2 medium. Organoids were then washed in DPBS and imaged in FluoroBrite™ DMEM (Gibco) on the Zeiss LSM700 confocal microscope.

### Methods of functional transport assay

Organoids were loaded in 1 mM 6-carboxyfluorescein (6CF) diluted in OptiMem for 35 min at 37 °C and then 10 mM of ethidium bromide was added to mixture for remaining 10 min. Organoids were then washed prior to imaging in OptiMem in a 30 mm coverglass bottom dish. A Leica SP8 confocal microscope with Okolab environmental chamber (37 °C and 5% CO_2_), motorized stage, and 25 × 0.95 N. A water immersion objective was used for live cell imaging. Conventional settings for fluorescein (488 nm laser line) and ethidium bromide (552 laser line) were used with sequential between line acquisition to avoid bleed through, bidirectional imaging for speed and 2 mm step size. At least ten well organized kidney organoids were imaged for each treatment group. Tubular structures were scored based on maximum projection views, cut through midsection from three different experiments. Experiments were carried out with MANZ2-2 iPSC line.

### Histochemistry and analysis

Kidney organoids fixed in 4% paraformaldehyde and embedded in paraffin as previously described [26]. Briefly, 6 µm thick sections were deparaffinized and heat-induced antigen retrieval performed using sodium citrate pH 6.0 buffer. Primary antibodies used were as follows; HAVCR1/KIM-1 (R&D Systems, AF1750), γH2AX (ThermoFisher, 50-194-123), HMOX-1 (Santa Cruz, sc-136960), nitrotyrosine (Novus, NB110-96877), Collagen 1a1 (Abcam, ab138492), Nephrin (PROGEN Biotechnik, GP-N2) CDH1 (BD Biosciences, 610182), Casp3 (BD Biosciences, 559565), KI67 (GenTex, GTX16667), MEIS1/2/3 (Antibodies-online, ABIN2668724). Collagen hybridizing peptide (CHP-Cy3, 3Helix) staining was performed after deparaffinization, according to manufacturer’s instructions. Fluorescently stained sections were imaged on a Zeiss LSM700 confocal microscope. All CHP and COL1A1 imaging was done under the same settings established on the no-hemin control. Analysis was performed using ImageJ by combining all of the single channel images into one stack, subtracting the background (rolling ball radius of 50.0 pixels, sliding parapoloid), and applying a threshold. Threshold was determined based on the controls for each assay, and subsequently applied to the stack. Area of the threshold was then measured and calculated by division of the DAPI threshold area value. For analysis at least 3 assays were examined with > 10 individual organoid sections per condition.

### Proteomic analysis

Approximately 100 organoids per sample (25 µg of 100 µg of total protein used per sample) were washed in cold PBS three times and flash frozen − 80 °C until processing. Samples were thawed on ice and resuspended in 1X SDS solubilization buffer (5% SDS in 50 mM TEAB), reduced by the addition of dithiothreitol (DTT) at a final concentration of 20 mM and heated to 95oc for 10 min. Alkylation was performed by the addition of iodoacetamide (IAA) to a final concentration of 40 mM followed by incubation in the dark for 30 min at room temperature. The SDS lysate was acidified with 12% aqueous phosphoric acid, 1:10 (v/v) to a final concentration of 1.2%. The solution was then diluted with the S-Trap protein binding buffer (90% methanol, 0.1 M TEAB, pH 7.55), 6:1 (v/v, S-Trap: total vol.). The mixture was transferred to the S-Trap™ micro spin column, centrifuged at 4,000 g for 20 s and processed according to manufacturer’s instructions. The three eluents were pooled and dried with vacuum centrifuging at 4 °C (Labconco, Kansas City, MO, USA). Peptides were re-suspended with 20 μL of 0.1% formic acid (FA) for fractionation. Fractionation was performed as described in the manufacturer’s manual (Cat No. 84868, Thermo Fisher Scientific). Desalted peptides were reconstituted in 0.1% FA and peptide concentration was determined using the Pierce™ BCA Protein Assay Kit. Samples were normalized to 0.5 µg/µL. Peptides were TMT-labeled as described previously [44]. TMT-tagged peptides were diluted to 300 μL with 0.1% trifluoroacetic acid. The two sets of 10 plex TMT tags were used to label peptides from a total of 18 samples. The samples were placed into separate batches with each experimental group (n = 3) represented in each batch. After quantification of the tagged peptides a pooled internal reference standard for each batch was made using equal volumes of each sample. The internal reference standard was used as the basis for determining the relative abundance of each experimental sample across the two different batches. The samples were run on a Thermo Scientific™ Orbitrap™ Tribrid™ mass spectrometer and analyzed with Proteome Discoverer™ Software. Experiment was carried out with MANZ2-2 iPSC line.

### Statistical analysis

Statistical significance was determined using one-way ANOVA, unpaired t with Welch’s correction in Prism (GraphPad). *P* values of < 0.05 were considered to be statistically significant.

## Results

### Hemin-induced injury in kidney organoids models kidney injury

Based on a previous studies, on which our organoid induction method is modeled, we hypothesized day 14 would be ideal for initiating hemin treatments, as the organoids are well formed at this stage and proliferation required to establish the nephrons has begun to diminish [26]. As the previously published protocol [26] was recently modified [27], we first confirmed that all the renal segment markers are expressed. We found that organoids contained the segmentation pattern as shown in previous protocols, with LTL and CDH1 labelling proximal and distal tubule respectively, NPHS1 labelling podocyte clusters and MEIS1/2/3 confirming presence of interstitial cells (Additional file 1: Fig. S1). Additionally, day 14 kidney organoids had low level of cells undergoing double-stranded breaks (γH2AX) and a proportion of the MEIS1/2/3 positive interstitial cells and tubules were proliferating (Additional file 1: Fig. S1). Since the day 14 organoids expressed the expected segment markers, we wanted to confirm if heme binding, and iron transporters were present to assay functional outcomes of hemin injured kidney organoids, therefore we performed both proteomic and RNA-Seq studies. We found day 14 kidney organoids expressed many heme pathway proteins necessary for homeostasis and hemolytic injury, including heme binding proteins (HEBP1, HEBP2), enzymes involved in catabolism of heme (BLVRA, BLVRB), iron transport (ACO1, LRP1, TFRC) and heme biosynthesis (ALAD, HMBS, UROD) (Additional file 1: Fig. S2). We also examined lower abundance, membrane-bound heme and iron transporters required for cellular heme and iron handling by RNA-Seq, and found expression of *LRP2, SLC40A1, HRG, SLC39A14, SLC11A2, SLC39A8* at day 14 (Additional file 1: Fig. S3) [45–47].

**Fig. 1:**
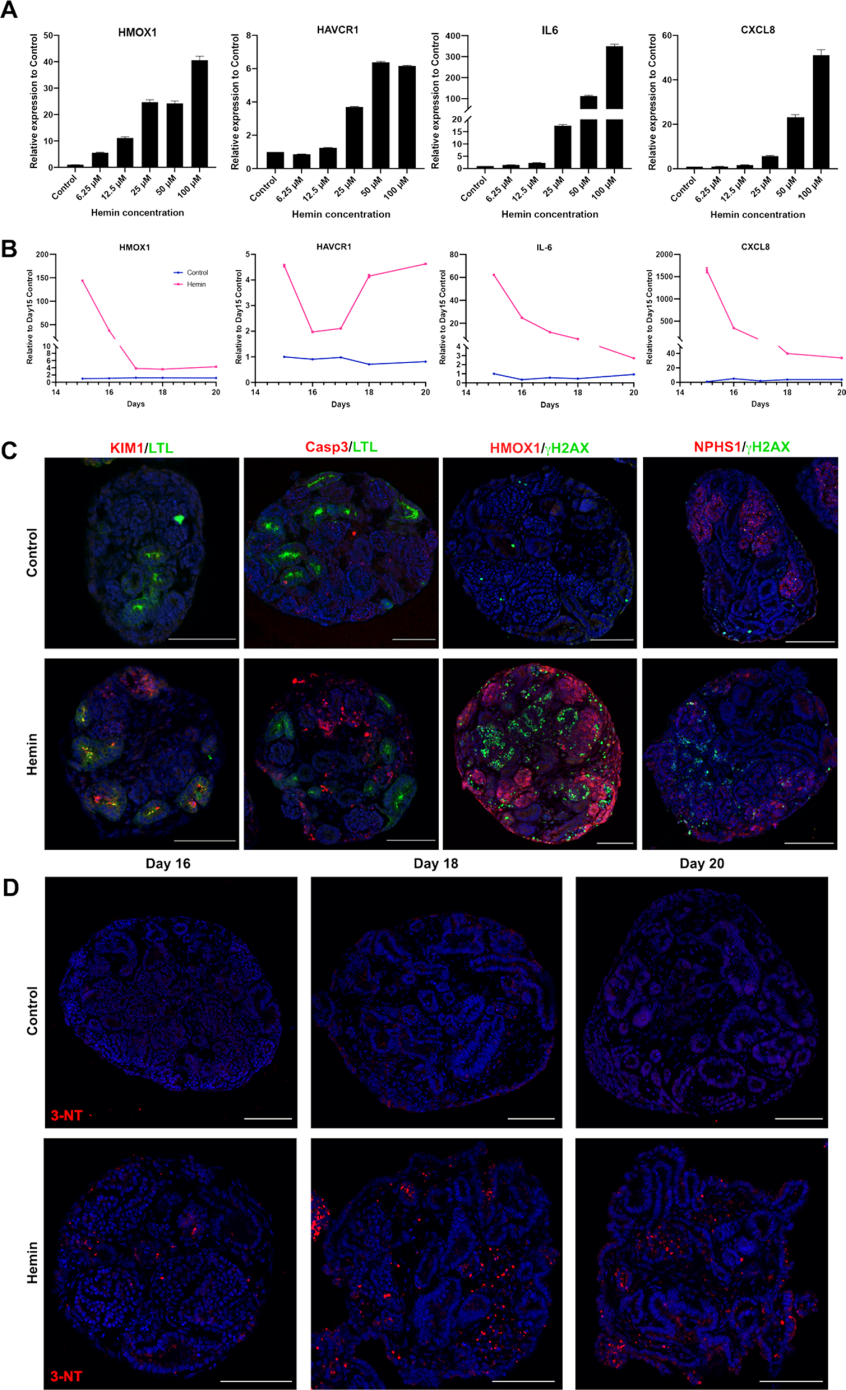
Hemin injury leads to induction of inflammation and nitric oxide mediated nitrosylation. **a** Representative quantitative PCR (qPCR) of kidney organoids treated with hemin at increasing concentrations. Expression relative to the control (untreated) organoids. **b** Time course qPCR of control and hemin treated organoids from day 15 to day 20. Results show mean and standard deviation of 3 technical replicates. **c** Immunofluorescence of kidney organoid paraffin sections of control and hemin treated organoids, showing localization of kidney injury marker 1 (KIM1, red) to proximal tubules labelled with Lotus tetragonolobus lectin (LTL, green), apoptotic marker activated caspase 3 (Casp3, red), DNA damage marker (γH2AX, green), heme catabolizing enzyme heme oxygenase 1 (HMOX1, orange), and nephrin labelling podocytes (NPHS1, red). **d** Immunofluorescence of kidney organoid paraffin sections at day 16, 18 and 20 showing 3-nitrotyrosine staining (red) indicative of nitrositive stress. Cell line MANZ2-2. Scale bar = 200 µm

Previous studies in cell culture monolayers have reported hemin dose treatments ranging between 10 and 30 µM lead to an increase in apoptosis and oxidative stress [48, 49]. To investigate whether hemin could be used to reproducibly induce injury in kidney organoids and recapitulate hemolytic events, we tested a range of hemin concentrations. Day 14 kidney organoids were treated with 6.25 µM to 100 µM hemin in a protein-free medium for 48 h. Following treatment, the extent of injury was examined by quantitative PCR for key inflammatory markers known to be upregulated during heme induced injury [50]. Induction of *HMOX1*, an enzyme that catabolizes hemin and is induced by ROS, showed a concentration-dependent and transient increase in gene expression (Fig. 1a; Additional file 1: Fig. S4A, B) indicative of a successful response to hemin treatment [51]. Expression of *HAVCR1* (a kidney injury marker, also known as KIM1) increased more than threefold with 25 µM of hemin, and more than a sixfold in concentrations of 50 µM and above, suggesting that proximal tubules in the kidney organoids were injured (Fig. 1a). A similar response was observed with the inflammatory markers *IL6* (17.4 fold at 25 µM, 113.3 fold at 50 µM, and 350 fold at 100 µM) and *CXCL8* (5.7 fold at 25 µM, 23.2 fold at 50 µM, and 51 fold at 100 µM). Based on these results we selected the 25 µM dose for further characterization as this was the lowest dose where a reproducible increase in all the injury/inflammatory markers was observed.

We next performed a time-course analysis for *HMOX1, HAVCR1, IL6* and *CXCL8* from day 15 (24 h post hemin addition) to day 20 of organoid culture (Fig. 1b; Additional file 1: Fig. S4C, D). Consistent with *HMOX1* being induced by ROS, its expression is maximal at day 15 (143.6 fold over control), and then sharply drops off once hemin is removed at day 16 (37.5 fold on day 16 and then low levels until day 20; Fig. 1b). This profile is roughly paralleled by *IL6* and *CXCL8* with a more gradual decline to day 20 (Fig. 1b; Additional file 1: Fig. S4C, D). Expression of *HAVCR1* shows a more complex profile with peaks of high expression relative to controls at day 15 and 20 (Fig. 1b). Immunofluorescence staining at these stages, as well as day 26, showed that hemin-treated organoids display high levels of HAVCR1 in the proximal tubules (labelled by Lotus tetragonolobus lectin; LTL) compared to the controls (Fig. 1c; Additional file 1: Fig. S5). We found that staining for γH2AX, a marker of double stranded DNA breaks that is induced by high levels of ROS, and caspase 3 (Casp3), labeling apoptotic cells, is also higher in the hemin-treated organoids compared to controls and was largely found in NPHS1-positive podocyte clusters (Fig. 1c). We next examined whether 3-nitrotyrosine, a marker of oxidative damage mediated by peroxynitrite, was increased in hemin treated organoids. We examined organoids treated at day 16, 18 and 20, and found very low levels of 3-nitrotyrosine in the control organoids. However, 3-nitrotyrosine was readily detected in hemin-treated organoids through day 20, where it appeared as punctate staining throughout the tissue, particularly in the stroma (Fig. 1d).

### Transcriptional analysis of hemin injury

**Fig. 2:**
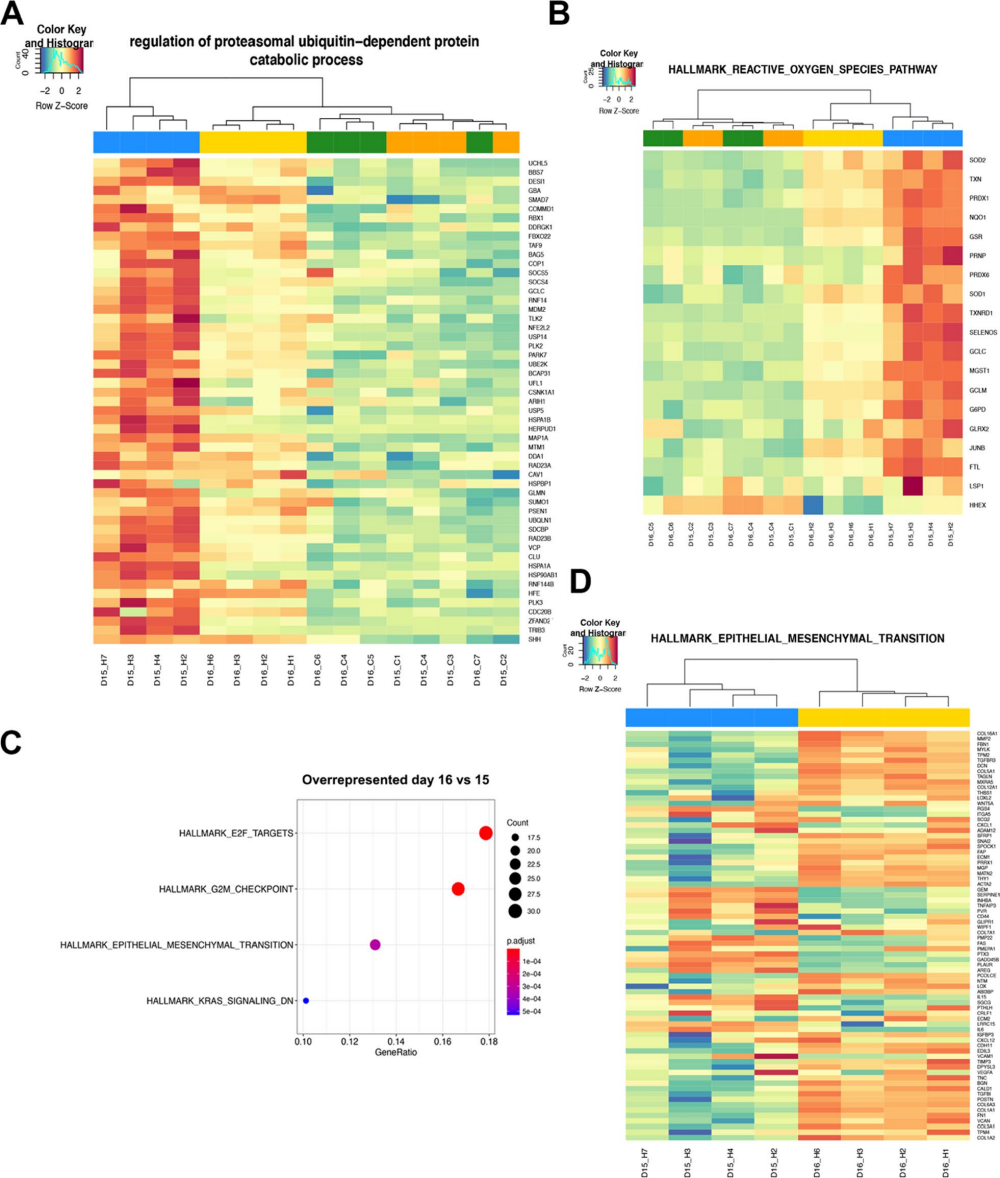
Transcriptome analysis of hemin injured kidney organoids. Heatmaps showing z-score of differentially expressed genes between untreated control day 15 and 16 (_C) and hemin treated kidney organoids (_H). **a** GO Proteasomal ubiquitin dependent protein catabolic process. **b** Hallmark pathway analysis reactive oxygen species pathway. **c** Dot plot of overrepresented Hallmark pathways between days 16 and 15. **d** Heatmap of Hallmark epithelial-to-mesenchymal transition pathway showing differences between hemin-treated organoids day 15 and 16. Cell line MANZ2-2

While the qPCR analysis of known injury markers demonstrated an injury response in hemin treated organoids, we wanted to assess transcriptional changes in response to hemin in a more comprehensive and unbiased fashion. To do this, we performed RNA-Seq on samples at days 15 and 16 (24 and 48 h post-hemin treatment, respectively; Fig. 2). Hallmark pathway analysis [52, 53] was performed to gain insight into the relevant biological processes being altered. The top pathways significantly upregulated at day 15 and 16 included those related to inflammation, apoptosis, and cellular stress (p53 pathway, hypoxia, UV response, unfolded protein response, reactive oxygen species; Additional file 1: Fig. S6). One major pathway that is induced in response to heme-induced stress is the protein degradation/ubiquitination pathway [54]. We found a number of proteasomal associated genes (*HSPA1B, HSBP1, HSPA1A, HSP90AB1*) and *UCHL5, UBQLN1*, *USP14* which are involved in the ubiquitination pathway [55], were significantly upregulated in the hemin-treated organoids on day 15 with lower, but still elevated, levels on day 16 (Fig. 2a). As expected, reactive oxygen species responsive genes (*SOD2, SOD1, NQO1*; Fig. 2b), and inflammatory markers *IL6*, *CXCL8*, *NFKB1*, *NFKBIA*, (Additional file 1: Fig. S7A) were also highly upregulated at day 15. We also examined differences between day 15 and 16 hemin treated samples to determine if EMT and collagen induction was taking place. The major pathways that were overrepresented at day 16 were associated with cell cycle and EMT in hemin treated organoids (Fig. 2, Additional file 1: Fig. S7B). In addition, a number of collagen and fibrosis related genes (*COL16A1*, *FBN1, COL5A1, COL12A1, ACTA2, COL1A1, COL6A3*) were upregulated at day 16 (Fig. 2d) suggesting initiation of early stages of fibrosis [56].

### Hemin injury leads to compromised function in the kidney organoids

**Fig. 3:**
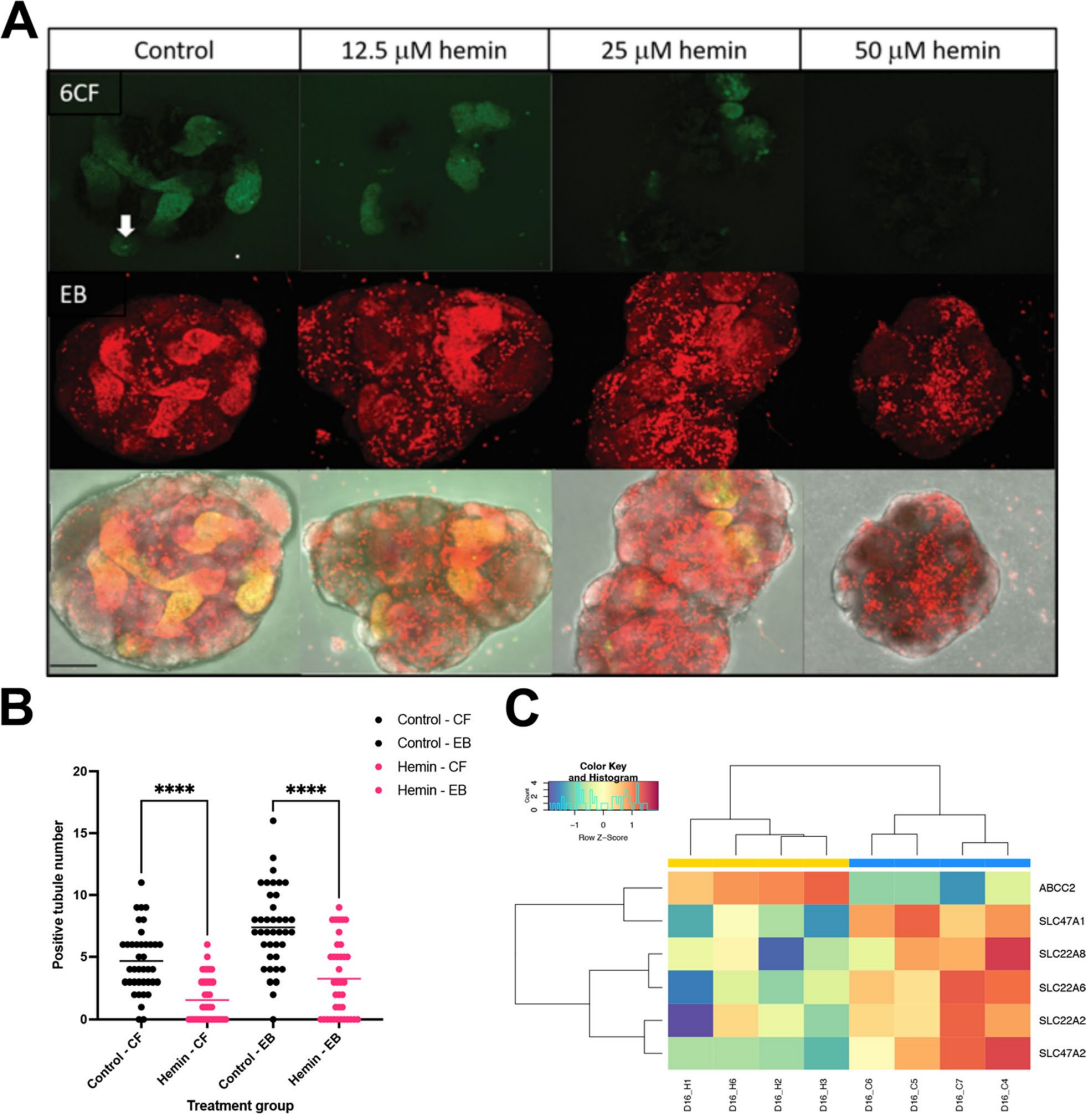
Assessment of proximal tubule transport function in kidney organoids. **a** Representative image of OAT (6CF) and OCT (EB) in the 12.5, 25 and 50 µM hemin treated kidney organoids. Note, transport declines with increasing concentrations of hemin. Each row shows the same corresponding organoid with 6CF, EB and overlay with transmitted light image. White arrow in 6CF control points to luminal accumulation in tubule structure. Maximum projections shown. Scale bar 100 µm. **b** Quantification of control vs 25 µM hemin treated kidney organoids showing changes between OAT (6CF) and OCT (EB) transport. Each dot represents one organoid from three independent assays and mean for each group is shown. One-way ANOVA with Dunnett’s T3 multiple comparisons test. ****P ≤ 0.0001. **c** Heat map showing expression in day 16 (D16) hemin (_H) and control (_C) samples of the OAT (SLC22A6, SLC22A8), OCT transporters (SLC22A2) and multi-drug resistant protein transporters (SLC47A1, SLC47A2, ABCC2). Cell line MANZ2-2

Since kidney organoids lack adequate vascularization and perfusion [25], transport throughout the nephron in the organoids is limited. However, we wanted to determine whether we could apply functional assays [57, 58] specifically adapted to test live kidney organoids and examine whether hemin-induced injury has an impact on organic anion and cation transport by the OAT and OCT transporters in the proximal tubule. We used a combined functional assay for proximal tubule transport of a fluorescent prototypical cation (ethidium bromide (EB)) and anion (6-carboxyfluorescein (6CF)) detected by fluorescence intensity and localization (e.g., luminal secretion or tubular uptake) (Fig. 3a). Kidney organoids were tested on day 16, after being treated for 48 h with hemin. We tested hemin concentrations of 12.5 µM, 25 µM, and 50 µM to confirm there was a concentration-dependent difference in function similar to what we observed with gene expression analysis (Figs. 1a, 3a). OAT function, as measured with 6CF, decreased with increasing concentrations of hemin, with essentially no detectable uptake or secretion at 50 µM hemin (Fig. 3a). OCT function, as measured with EB, showed decreased tubular fluorescence at 50 µM hemin, and an increase in punctate fluorescence representative of EB intercalating into the double-stranded breaks in the DNA, marking apoptotic cells, throughout the kidney organoids at all doses (Fig. 3a) [59]. RNA-Seq analysis of specific basolateral (*SLC22A2*, *SLC22A6*, *SLC22A8*) and apical transporters (*SLC47A1*-*A2, ABCC2*) associated with organic anion and cation transport showed that they were all downregulated in the hemin-treated samples, except for *ABCC2*, (coding for a multidrug resistant protein 2 – MRP2) which was higher in the hemin-treated samples. These results suggest that overall reduction in transport is associated with reduced gene expression of proximal tubule transporters, likely because of hemin-induced cellular injury. Additionally, the increase in *ABCC2* may suggest a greater cellular detoxification effort [60].

### Cytochrome-C-GFP biosensor to model injury in real-time

**Fig. 4:**
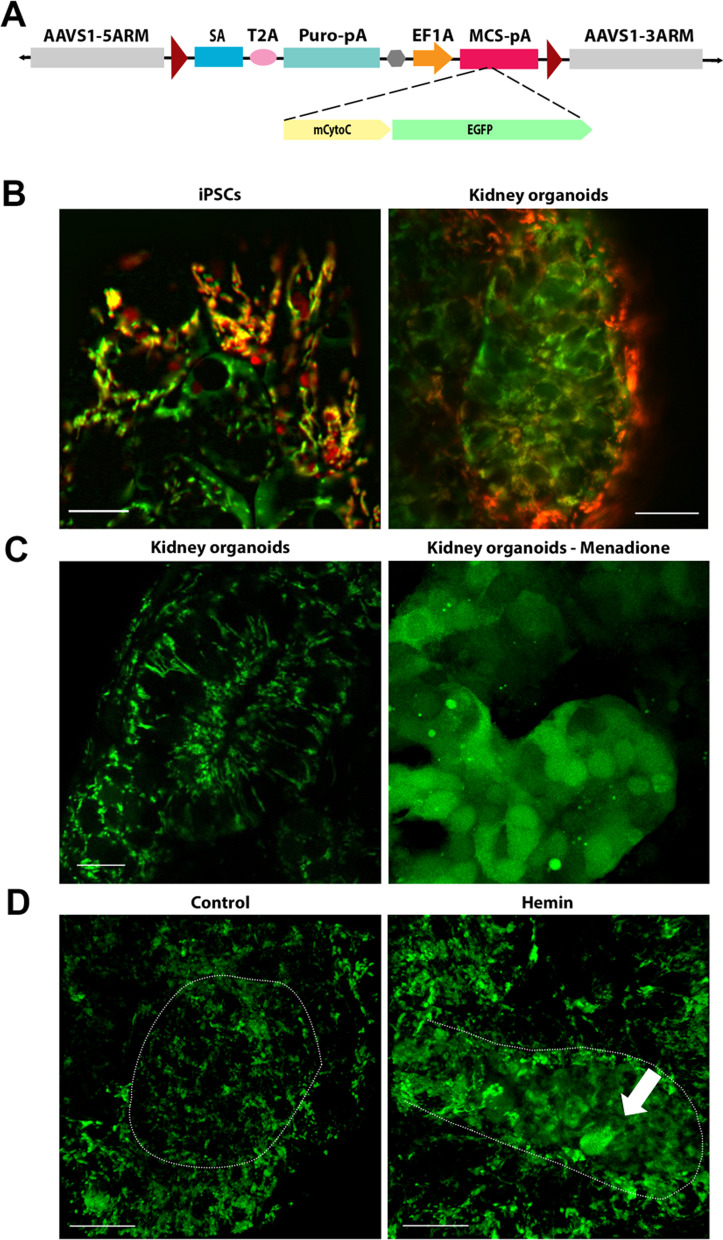
CytoC-GFP biosensor localizes to the mitochondria. **a** Schematic representation of the safe harbor AAVS1 donor vector with CytoC-GFP construct. **b** Live immunofluorescence showing CytoC-GFP expression is colocalized to MitoTracker Red CMXRos labelled mitochondria in the iPSCs and kidney organoids. Mitochondrial expression in the tubule of the kidney organoids, and diffuse cytoplasmic staining in the menadione treated tubule. Scale bar = 10 µm. **c** Representative live images of 3D projections showing mitochondrial localization in the control and 25 µM hemin treated tubules of kidney organoids. Note the diffuse CytoC-GFP localization in the hemin treated tubule (white arrow). Data from isolated clone #8 CytoC-GFP iPSC line. Scale bars = 20 µm

For kidney organoids to be useful as a platform to screen drug toxicity or to treat renal injury, it would be ideal to assess early signs of apoptosis and injury via non-invasive, real-time monitoring. Towards this end we generated iPSC reporter lines that express CytochromeC-GFP (CytoC-GFP), a biosensor that is released from mitochondria upon cellular injury to assess apoptosis [15]. In healthy non-apoptotic cells, CytoC-GFP is associated with mitochondria and shows a punctate or network-like fluorescence pattern. Upon cellular injury or increase in ROS, the fluorescence pattern rapidly shifts to being cytoplasmic. We confirmed that the CytoC-GFP signal in iPSCs was colocalized to the mitochondria by live co-immunofluorescence with MitoTracker Red CMXRos, a mitochondrial membrane potential dependent dye (Fig. 4b, Additional file 1: Fig. S8). Next, we generated kidney organoids from the CytoC-GFP iPSCs and treated them at day 14 with menadione, known to depolarize the mitochondrial membrane and induce apoptosis [61]. Menadione treated organoids exhibited diffuse cytoplasmic staining of CytoC-GFP in comparison to the untreated control (Fig. 4b, Additional file 1: Fig. S8). To confirm that inserting the CytoC-GFP transgene into the MANZ2-2 cell line did not increase the basal levels of ROS and apoptosis, we performed live staining with a superoxide indicator, dihydroethidium, which showed comparable levels of nuclear staining between the MANZ2-2 and CytoC-GFP cell lines (Additional file 1: Fig. S9A). Furthermore, levels of Casp3 were also comparable between the two cells lines (Fig. 1c, Additional file 1: Fig. S9B). To examine the effect of hemin treatment, CytoC-GFP kidney organoids were monitored over a 48-h window, post-hemin treatment. While control organoids showed CytoC-GFP signal in discrete elongated and interconnected tubular structures, consistent with mitochondria, hemin treatment induced foci of cells within the tubules and podocyte clusters with diffuse CytoC-GFP signal (Fig. 4c). These results suggest hemin-induced ROS is driving apoptotic events, which are leading to mitochondrial dysfunction.

### Hemin-injured organoids show reduced fibrosis upon treatment with UPHD25

**Fig. 5:**
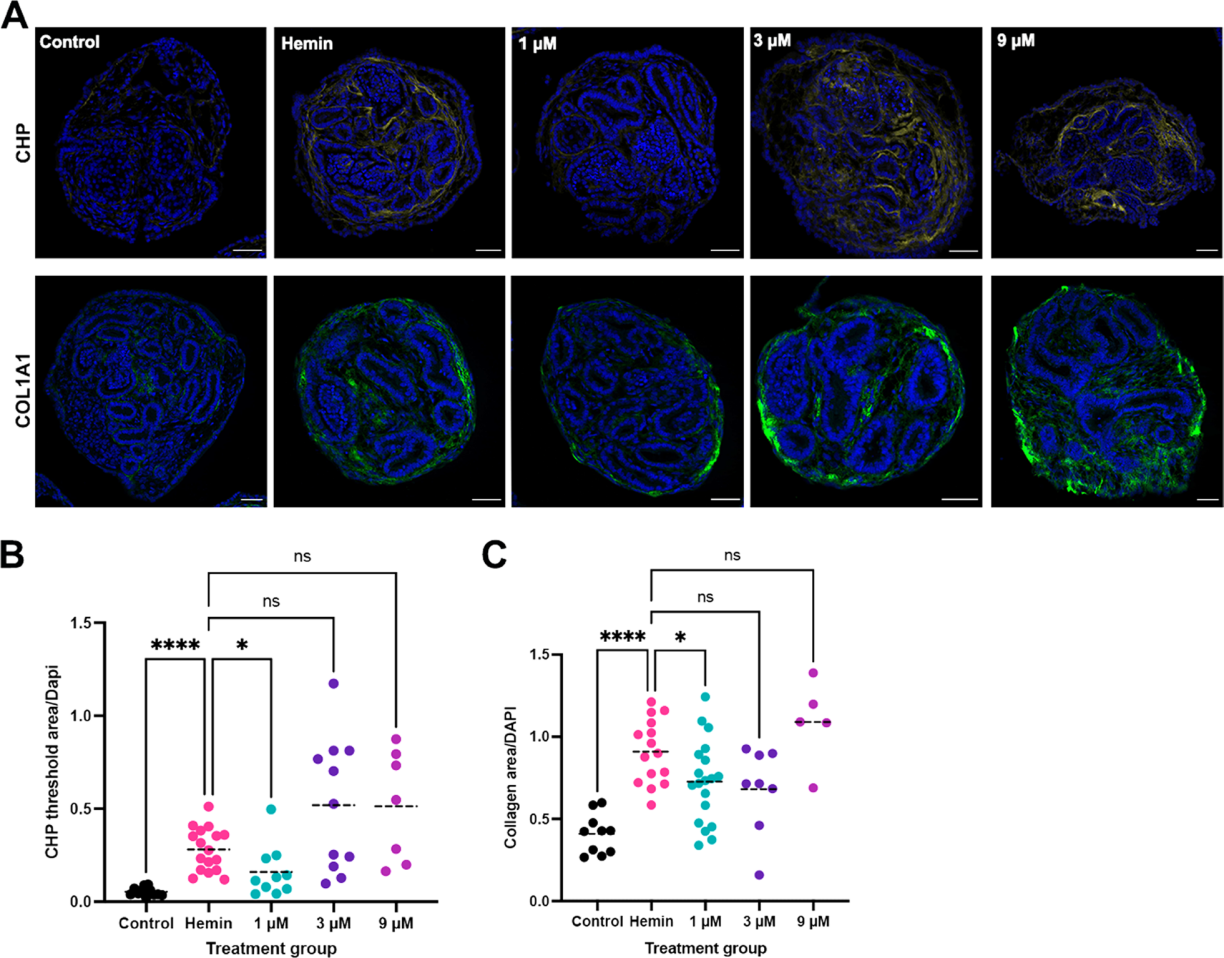
Treatment with UPHD25 reduces collagen induction in kidney organoids. **a** Representative images of paraffin sections of kidney organoids at day 26, stained with collagen hybridizing peptide (CHP; yellow) and collagen 1a1 antibody (COL1A1; green), DAPI staining nuclei (blue). UPHD25 concentrations 1, 3, and 9 µM. **b** Quantification of CHP staining. Each point represents CHP threshold area/DAPI per organoid, per section. **c** Quantification of COL1A1 staining. Each point represents COL1A1 threshold area/DAPI per organoid, per section. One-way ANOVA with multiple comparisons to hemin control. ns, non-significant; *P ≤ 0.05; ****P < 0.0001. Cell line MANZ2-2. Scale bar = 100 µm

ROS induced inflammation in the kidney leads to an upregulation of pro-inflammatory and pro-fibrotic cytokines, such as MCP1 and TGF-β1, resulting in interstitial fibrosis [50]. We therefore tested if hemin treatment increased deposition of collagen in kidney organoids. Following a 48-h hemin treatment, hemin was washed out and kidney organoids were cultured to day 26. Based on previously published data [26], we know there is significant collagen deposition by day 26, however with modifications of the protocol [27] there is less collagen seen at day 26, thus allowing us to compare treated and untreated conditions at this timepoint. To determine the extent of the fibrotic response, we stained kidney organoid sections with collagen hybridizing peptide (CHP) which stains collagen undergoing remodeling or degradation seen in fibrotic diseases, and has been shown to be an accurate measure of organ fibrosis [62–64]. We found that there was a significant increase in CHP staining in hemin-treated kidney organoids compared to controls (Fig. 5a, b; Additional file 1: Fig. S10A, B). Furthermore, we performed staining with collagen 1a1 (COL1A1) and found the trend was comparable to CHP data (Fig. 5a, b; Additional file 1: Fig. S10A, B). To determine if the hemin injury model is responsive to therapeutic intervention, we examined whether the fibrotic response could be pharmacologically inhibited. In prior murine studies, we have shown that 4-(phenylthio)butanoic acid (PTBA) displays anti-fibrotic activity when delivered as a prodrug (UPHD25 or UPHD186) [29, 31]. We therefore tested the effect of the methyl ester PTBA prodrug UPHD25 in hemin-injured kidney organoids. We tested doses of 1, 3 and 9 µM of UPHD25 in the hemin injury assay by delivering daily treatments from days 16–26 following hemin treatment from days 14–16. We tested two independent cell lines (MANZ2-2 (female origin) and MANZ4-37 (male origin)) and found that UPHD25 decreased CHP and COL1A1 staining at a concentration of 1 µM in MANZ2-2 cell line, and decreased CHP staining with 3 and 9 µM in MANZ4-37 (Fig. 5b, c. Additional file 1: Fig. S10B, C). These results suggest the fibrotic response in kidney organoids can be reduced with therapeutic intervention.

## Discussion

Kidney organoids can be generated in large quantities, genetically manipulated, and have been shown to be a biologically relevant model for human disease [25]. Producing kidney organoids in bulk makes them well suited for testing therapeutic interventions [26]. While shortcomings with kidney organoid maturity are well-documented, [23] they are a good system to develop models for chronic organ diseases and fibrosis. Liver and lung organoids, which also display fetal like states comparable to early trimester development, have been used to study pulmonary and liver fibrosis [65].

Hemin is a potent inducer of ROS that mimics the hemolytic response seen in many diseases leading to renal injury [3]. Time course analysis of hemin-treated organoids revealed that the response to hemin injury was immediate and acute, with the inflammatory response and *HMOX1* induction being rapidly downregulated within a couple of days of hemin removal, which is consistent for *HMOX1* expression in injury settings [51]. However, sustained *HAVCR1* expression suggests that tubule cells undergo enduring injury in response to the brief hemin exposure. This is in accordance with other hemolytic models in mice which show a rapid decline of inflammatory markers but persistent expression of *HAVCR1* [66]. Unilateral urothelial obstruction (UUO) and IRI injury models in mice also show that continued *HAVCR1* expression is associated with higher levels of fibrosis and a progression to CKD [67, 68]. Elevated perdurance of HAVCR1 also translates to the clinic as not only do patients suffering from acute injury show elevated levels of urinary HAVCR1, but patients suffering from chronic injury show even higher levels of HAVCR1 [69].

High levels of ROS and nitrogen species are found during hemolytic events forming highly reactive peroxynitrite, and lead to nitrosylation of proteins [14]. We found that 3-nitrotyrosine (byproduct of nitration) was elevated in hemin treated organoids. In a condition unrelated to hemin induced injury, patients with septic shock and CKD show high levels of nitrotyrosine as well as nitrite/nitrate (indicating NO production) levels in plasma, suggesting the nitration of tyrosine residues is directly related to disease causing events [70]. In addition, rats loaded with iron exhibited nitrotyrosine labeled tubules and interstitial cells. They had elevated levels of NO synthase enzymes (responsible for NO production), indicating increased levels of reactive nitrogen species due to high levels of iron [71]. This work supports the notion that high peroxynitrite levels and nitric oxide levels can play a critical role in hemolytic events and the subsequent injury. Another major cellular response to high oxidative stress is an increase in ubiquitination and proteasomal degradation of proteins [72]. Our RNA-Seq analysis revealed the top differentially expressed genes were part of the ubiquitin–proteasome degradation network and one of the overrepresented pathways was the unfolded protein response, suggesting the hemin treated organoids were trying to eliminate unfolded or damaged proteins in order to ameliorate cellular injury [73].

Inhibition of the proteasome system leads to an increase in ubiquitination of mitochondrial proteins and recruitment of heat shock, and autophagy related components [74]. Therefore, mitochondria need to be continuously monitored during ROS events for potential dysfunction. We developed the CytoC-GFP iPSC line as a real-time biosensor, which can be utilized to monitor mitochondrial health, morphology, and cellular response to toxins [15]. Recent studies show that CytoC may have other functions beyond apoptosis induction, which could further aid in understanding injury mechanisms. CytoC has been shown to translocate to the nucleus before activating the apoptotic cascade, where the protein plays a role in the DNA damage repair response [75, 76]. This suggests that not all cells that release CytoC from the mitochondria undergo apoptosis. Dissecting this mechanism further, may shed light on the repair pathways and potential future treatments. Integration with new imaging technologies and other biosensors may further our understanding of cellular injury particularly in PT cells which are rich in mitochondria [77].

Finally, we show that our model of hemin mediated injury can be adapted to therapeutic compound testing by showing a reduction in the fibrotic response. Previous studies have shown that PTBA, delivered as a prodrug, can ameliorate injury and reduce fibrosis in multiple models of renal injury [29–31]. As with most potential renal therapeutics, PTBA has never been validated against a human proteome, especially in an injury setting. In addition, when performing therapeutic injury studies, several shortcomings need to be considered. Kidney organoids are immature and are more closely related to embryonic kidney epithelium and interstitium [78]. In addition, longer-term injury studies are currently not feasible, as organoids lack a blood supply, filtration, or tubular flow, and are lacking an immune system, which may not allow modeling of some of the molecular and cellular responses seen in injured adult kidneys [79]. However, organoids still have the potential to be useful for studying the efficacy and safety of potential therapeutics, as they provide the advantage of multiple cell types and structures over standard 2D culture systems [80].

## Conclusions

This work demonstrates we can model aspects of renal injury such as fibrosis and pro-inflammatory cytokine expression, and therapeutically intervene to reduce the injury and possibly stimulate repair. For the scientific community to take full advantage of kidney organoid systems, we need to further our understanding of the injury processes that take place in organoids and how they compare to in vivo kidney injury*.* This includes understanding the repair process that occurs after the initial insult and the longer-term outcomes. The hemin injury model displays many of the same hallmarks as in vivo hemolytic injury, and may allow validation of the efficacy of targeted renal therapeutics in a human model of renal injury. However, as with any preclinical model there are limitations, and while this model maybe a good model of cellular injury, due to the limitations of the kidney organoids it cannot model other hemin mediated injury events such as tubular obstruction or vasoconstriction.

## Supplementary Information

**Additional file 1.** Supplementary Figures.

## Data Availability

All relevant data have been uploaded as part of supplementary files and available on National Center for Biotechnology Information’s GEO database.

## Abbreviations

6CF: 6-Carboxyfluorescein
ACO1: Aconitase 1
ALAD: Aminolevulinate dehydratase
AKI: Acute kidney injury
BLVR: Biliverdin reductase
CHP: Collagen hybridizing peptide
CKD: Chronic kidney disease
COL_A_: Collagen Type_Alpha_
CXCL8: C-X-C Motif Chemokine Ligand 8
CytoC-GFP: Cytochrome C-GFP
DE: Differential expression
EB: Ethidium bromide
FBN1: Fibrillin 1
FA: Formic acid
HAVCR1: Hepatitis A virus cellular receptor 1
HDAC: Histone deacetylase
HEBP: Heme binding protein
HMBS: Hydroxymethylbilane synthase
HMOX1: Heme oxygenase 1
HRG: Histidine rich glycoprotein
IL6: Interleukin 6
iPSCs: Induced pluripotent stem cells
IRI: Ischemia reperfusion injury
LRP: LDL receptor related protein
LTL: Lotus tetragonolobus lectin
MCP1: Monocyte chemoattractant protein-1
MRP2: Multidrug resistant protein 2
NO: Nitric oxide
NQO1: NAD(P)H quinone dehydrogenase 1
OAT: Organic anion transporter
OCT: Organic cation transporter
PTBA: 4-(Phenylthio) butanoic
ROS: Reactive oxygen species
SLC_: Solute Carrier Family_Member
SOD: Superoxide dismutase 1
TFRC: Transferrin receptor
TGF- β1: Transforming growth factor beta 1
UCHL5: Ubiquitin C-terminal hydrolase L5
UBQLN1: Ubiquilin 1
USP14: Ubiquitin specific peptidase 14
ULA: Ultra-low attachment
UROD: Uroporphyrinogen decarboxylase
UUO: Unilateral urothelial obstruction
