# Supplementary material for: Modeling oxidative injury response in human kidney organoids
Source: Stem Cell Res Ther. 2022 Feb 21;13:76. doi: 10.1186/s13287-022-02752-z (PMC8862571; doi:10.1186/s13287-022-02752-z)
Supplement: Supplementary file 1 — Additional file 1. Supplementary Figures. [file 13287_2022_2752_MOESM1_ESM.docx]

**Modeling oxidative injury response in human kidney organoids**

**Supplemental data**


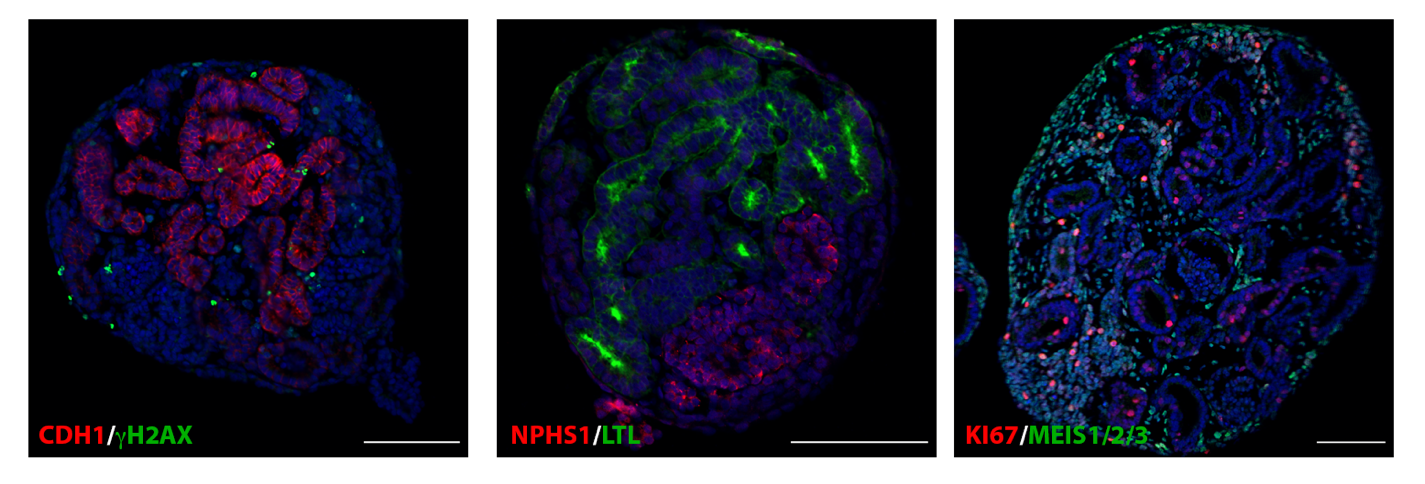


**Supplemental Figure 1: Day 14 segment marker analysis**

Immunofluorescence of paraffin sectioned day 14 kidney organoids stained with Lotus tetragonolobus lectin (LTL) labelling proximal tubule segment, Cadherin 1 (CDH1) labelling distal tubule segment, nephrin (NPHS1) labelling podocytes, γH2AX labelling cells undergoing DNA damage, KI67 labelling proliferating cells and MEIS1/2/3 labelling renal interstitial cells. Scale bar = 100 µM

**Supplemental Figure 2. Heme pathway and iron transport protein abundance in organoid assays.**

Relative abundance of proteins that were identified in all three separate kidney organoid assays. Abundance was calculated from the total peptides vs a pooled internal reference standard. Each dot represents one assay. ALAD, Aminolevulinate dehydratase; BLVRA, Biliverdin reductase A; BLVRB, Biliverdin reductase B; HEBP1, Heme binding protein 1; HEBP2, Heme binding protein 2; HMBS, Hydroxymethylbilane synthase; LRP1, LDL receptor related protein 1; UROD, Uroporphyrinogen decarboxylase; HEPH, Hephaestin; TFRC, Transferrin receptor; ACO1, Aconitase 1; CAND1, Cullin associated and neddylation dissociated 1; FTL, Ferritin light chain; GLRX, Glutaredoxin; LTF, Lactotransferrin.

**Supplemental Figure 3. RNA-seq analysis of day 14 control organoids.**

Average FPKM four separate kidney organoid samples. OSR1, Odd-skipped-related 1; LRP2, Low density lipoprotein-related protein 2; SLC40A1, Ferroportin-1; HRG, Histidine-rich glycoprotein; SLC39A8, Metal Cation Symporter ZIP8; SLC22A1, Organic Cation Transporter 1; SLC22A2, Organic Cation Transporter 2; SLC22A3, Organic Cation Transporter 3; SLC39A14, Metal Cation Symporter ZIP14; EYA1, Eyes Absent Homolog 1; SLC22A6, Organic Anion Transporter 1; SLC22A8, Organic Anion Transporter 2; PAX2, Paired Box 2; SLC11A2, Divalent Cation Transporter 1; SIX1, Sine Oculis Homeobox Homolog 1; LHX1, LIM Homeobox 1; BMP7, Bone Morphogenic Protein 7.


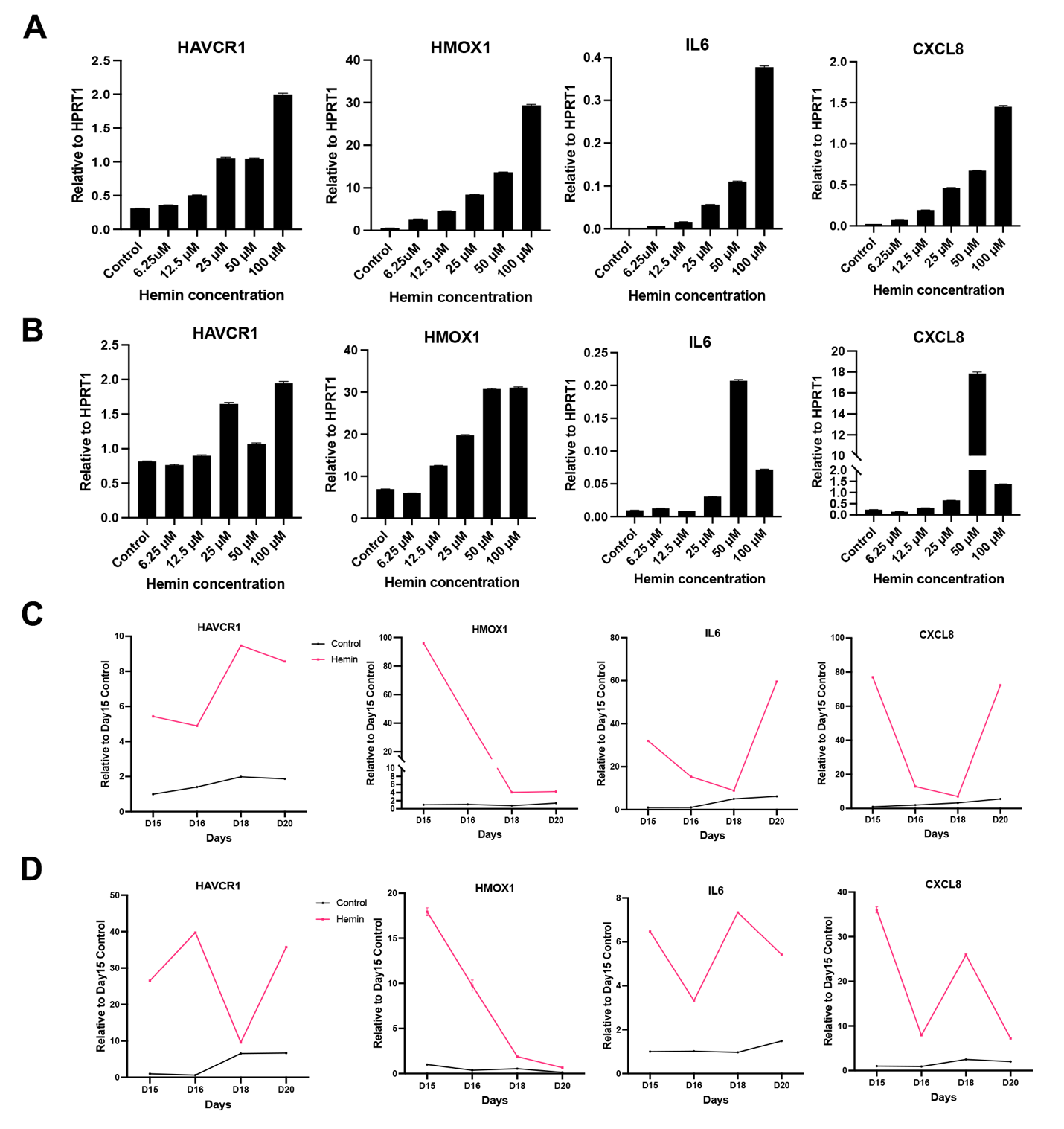


**Supplemental Figure 4. Hemin injury leads to induction of injury markers.**

Quantitative PCR (qPCR) of kidney organoids treated with different concentrations of hemin (A-B). Time course qPCR analysis of organoids treated with 25 µM hemin (C-D). Figures A and C are MANZ2-2, B and D are MANZ4-37 kidney organoid assays.


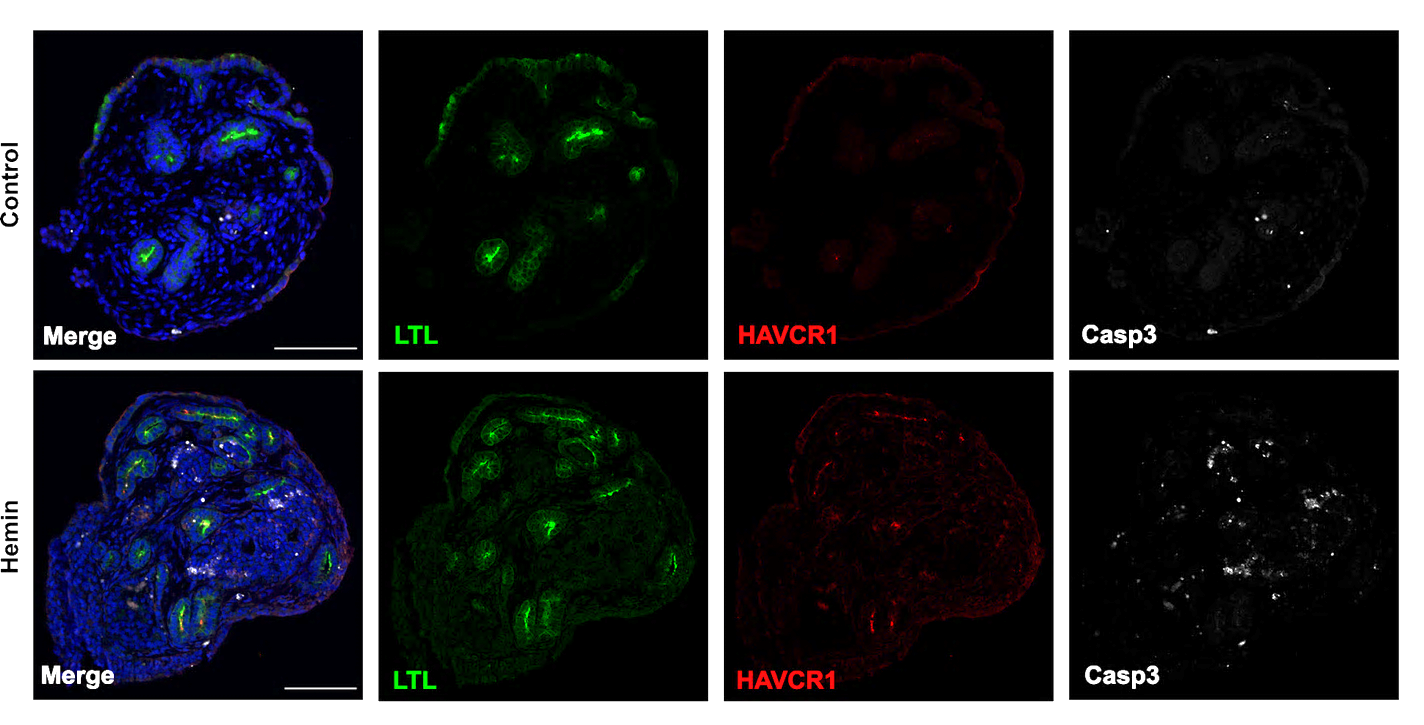


**Supplemental Figure 5. Day 26 hemin injured kidney organoids.**

Immunofluorescence of paraffin sectioned day 26 kidney organoids stained with Lotus tetragonolobus lectin (LTL) labelling proximal tubule segment, HAVCR1 (also KIM1) labelling injured proximal tubule cells (yellow arrows), and apoptotic cells labelled with Casp3. Scale bar = 200 µM


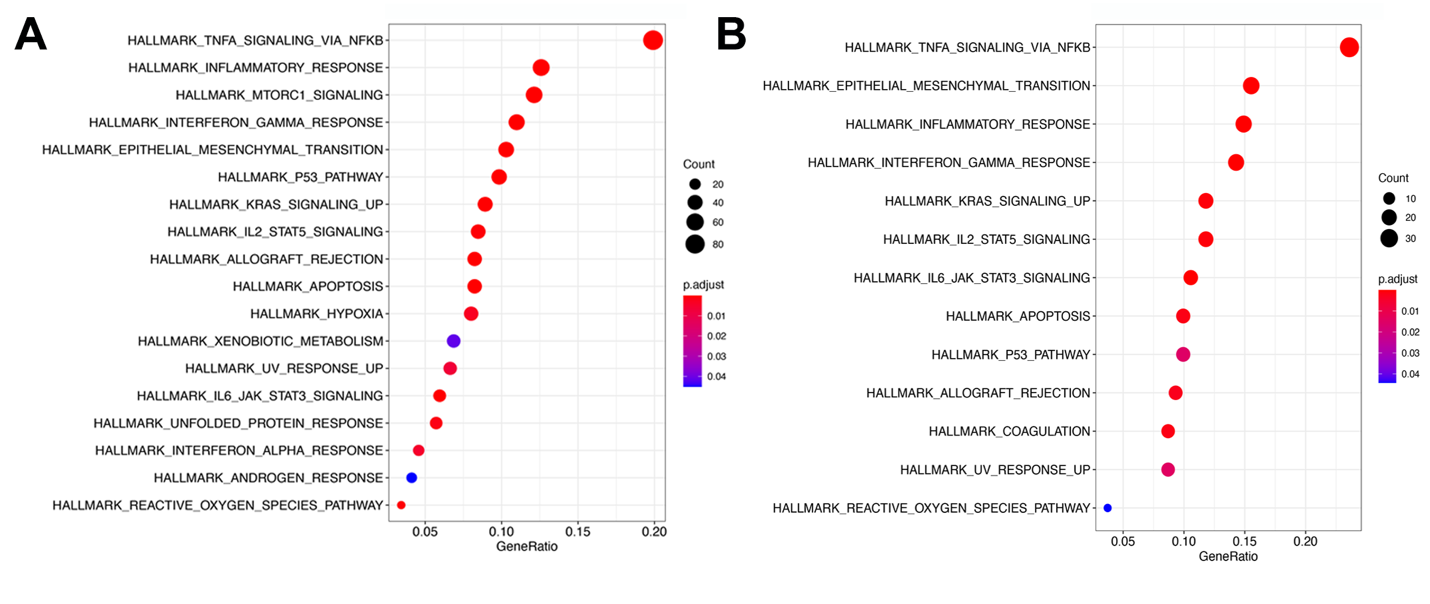


**Supplemental Figure 6. Hallmark pathway enrichment analysis**

Dot plots showing most significant upregulated pathways of hemin treated kidney organoids at **A)** day 15 (24 hrs hemin treatment) and **B)** at day 16 (48 hrs hemin treatment). The dot size represents the number of genes enriched in the specific Hallmark pathway. The color represents the statistical significance level of specific Hallmark pathway enrichment.


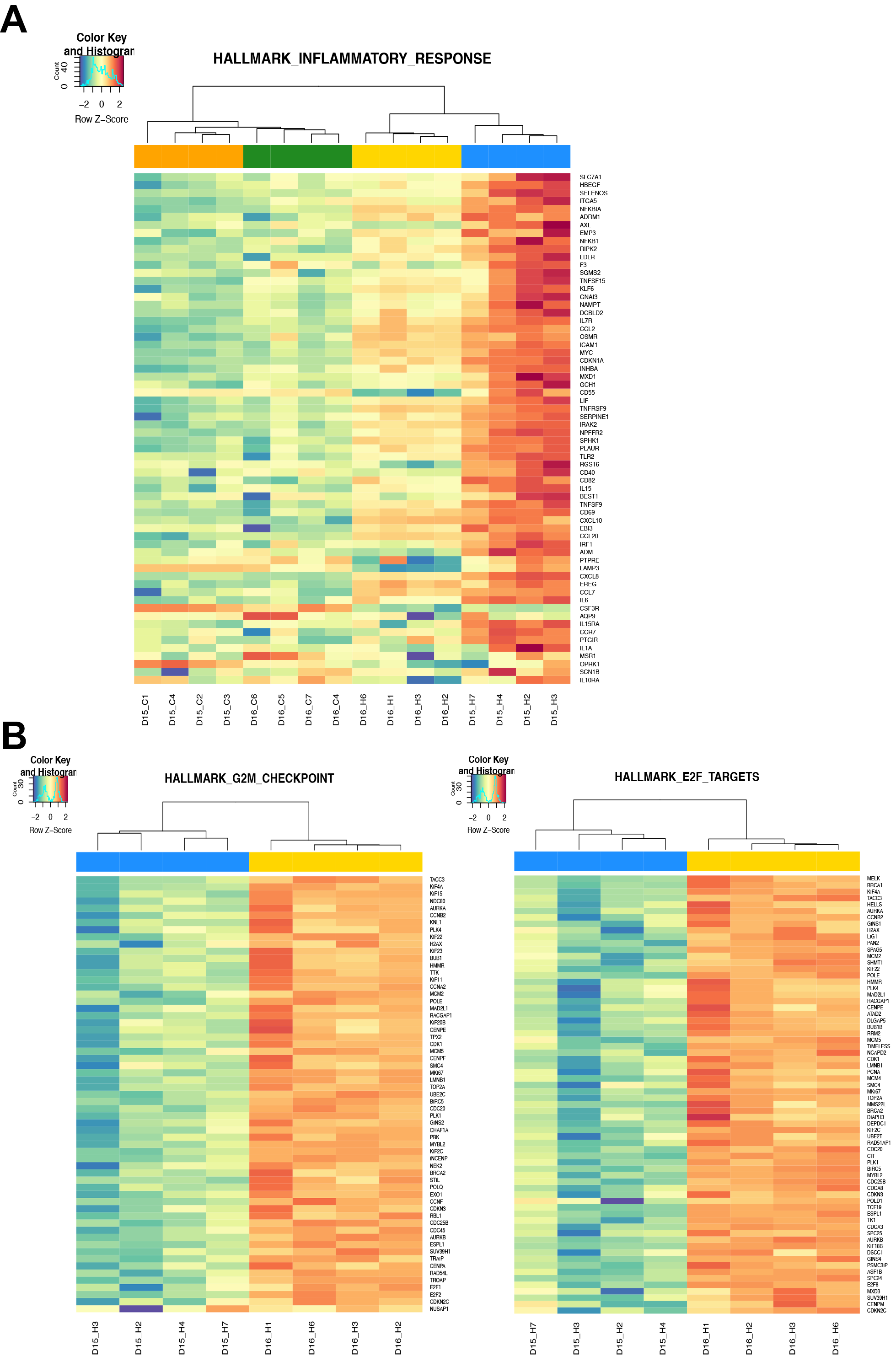


**Supplemental Figure 7. Hallmark pathway analysis**

Heatmaps showing z-score of differentially expressed genes between untreated control day 15 and 16 (_C) and hemin treated kidney organoids (_H). **A)** Hallmark pathway analysis of inflammatory response. **B)** Hallmarks pathway analysis showing difference between day 15 and 16 hemin-treated organoids, G2M checkpoint pathway and E2F pathway.


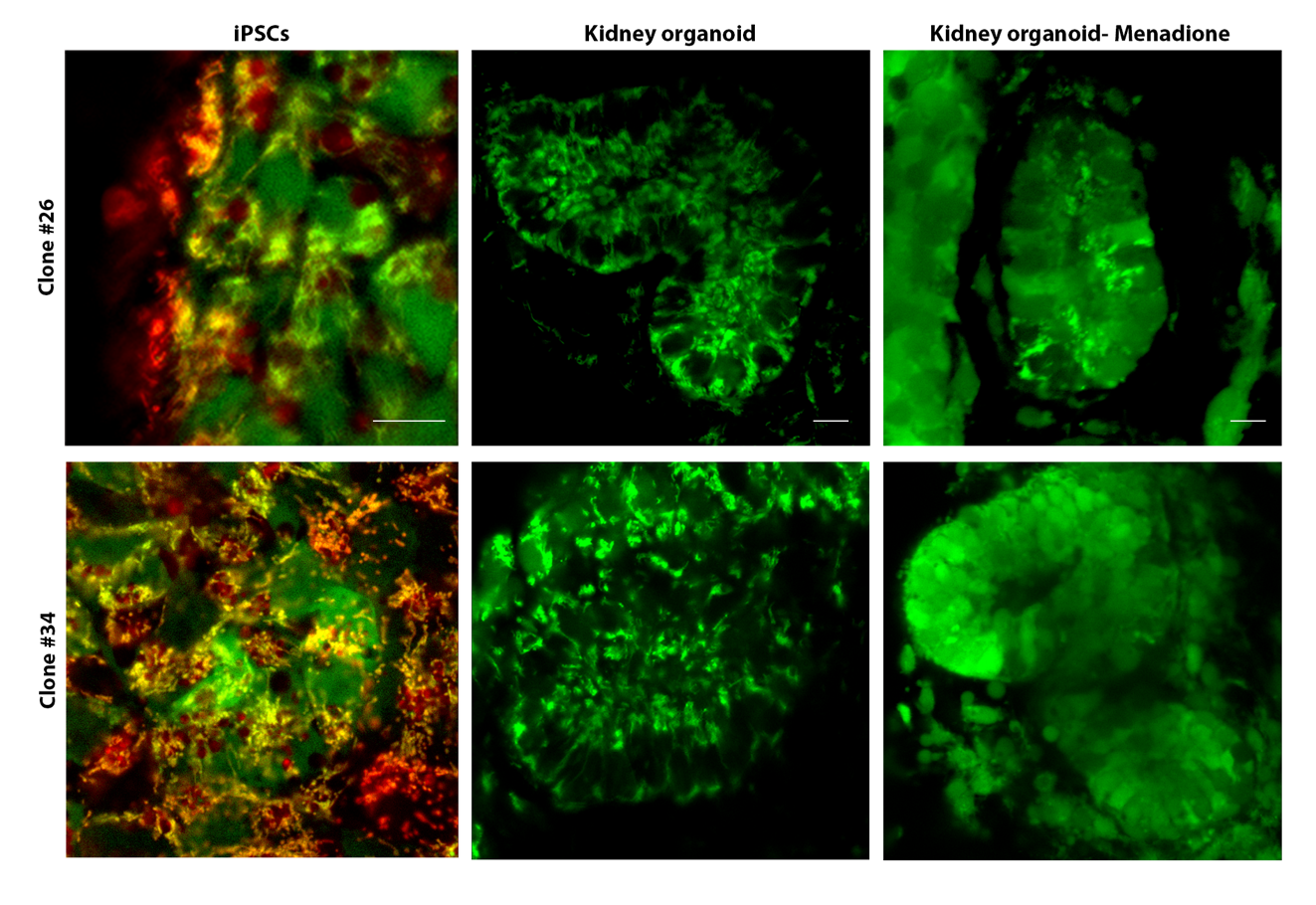


**Supplemental Figure 8. CytoC-GFP biosensor localizes to the mitochondria**

Live immunofluorescence showing CytoC-GFP expression is colocalized to MitoTracker Red CMXRos labelled mitochondria in the iPSCs in two isolated clones. Mitochondrial expression in the tubule of the kidney organoids, and diffuse cytoplasmic staining in the menadione treated tubule. Scale bars = 10 µm**.**


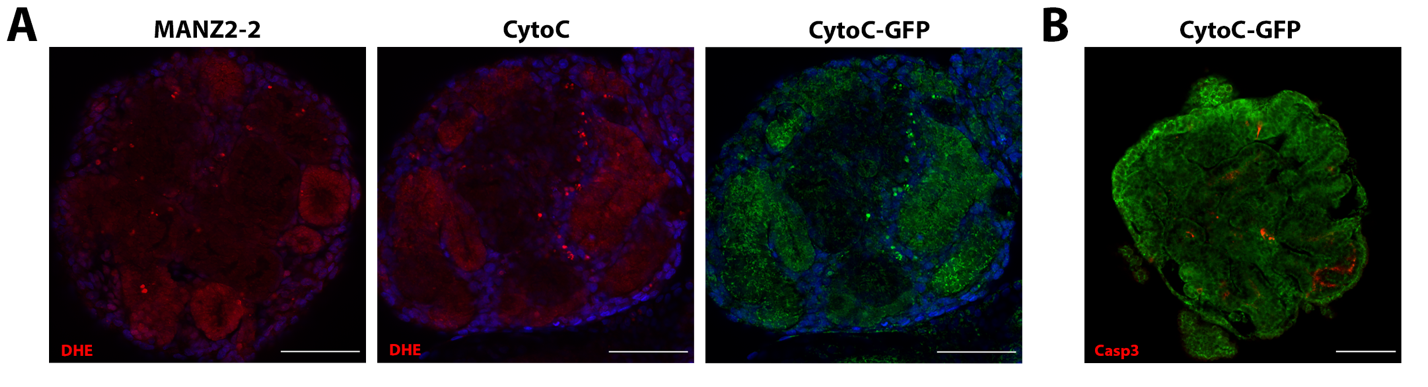


**Supplemental Figure 9. CytoC-GFP biosensor levels of ROS and apoptosis**

Immunofluorescence of MANZ2-2 and CytoC-GFP#8, 14 kidney organoids. A) Live staining with superoxide indicator dihydroethidium (DHE) labelling positive nuclei in both MANZ2-2 and CytoC-GFP#8 organoids as a comparison. Blue = nuclei. Scale bars = 200 µM. Note: cytoplasmic staining is not indicative of positive DHE cell. B) Fixed and immunolabelled CytoC-GFP#8 kidney organoid with apoptosis marker Casp3. Scale bar = 100 µM.


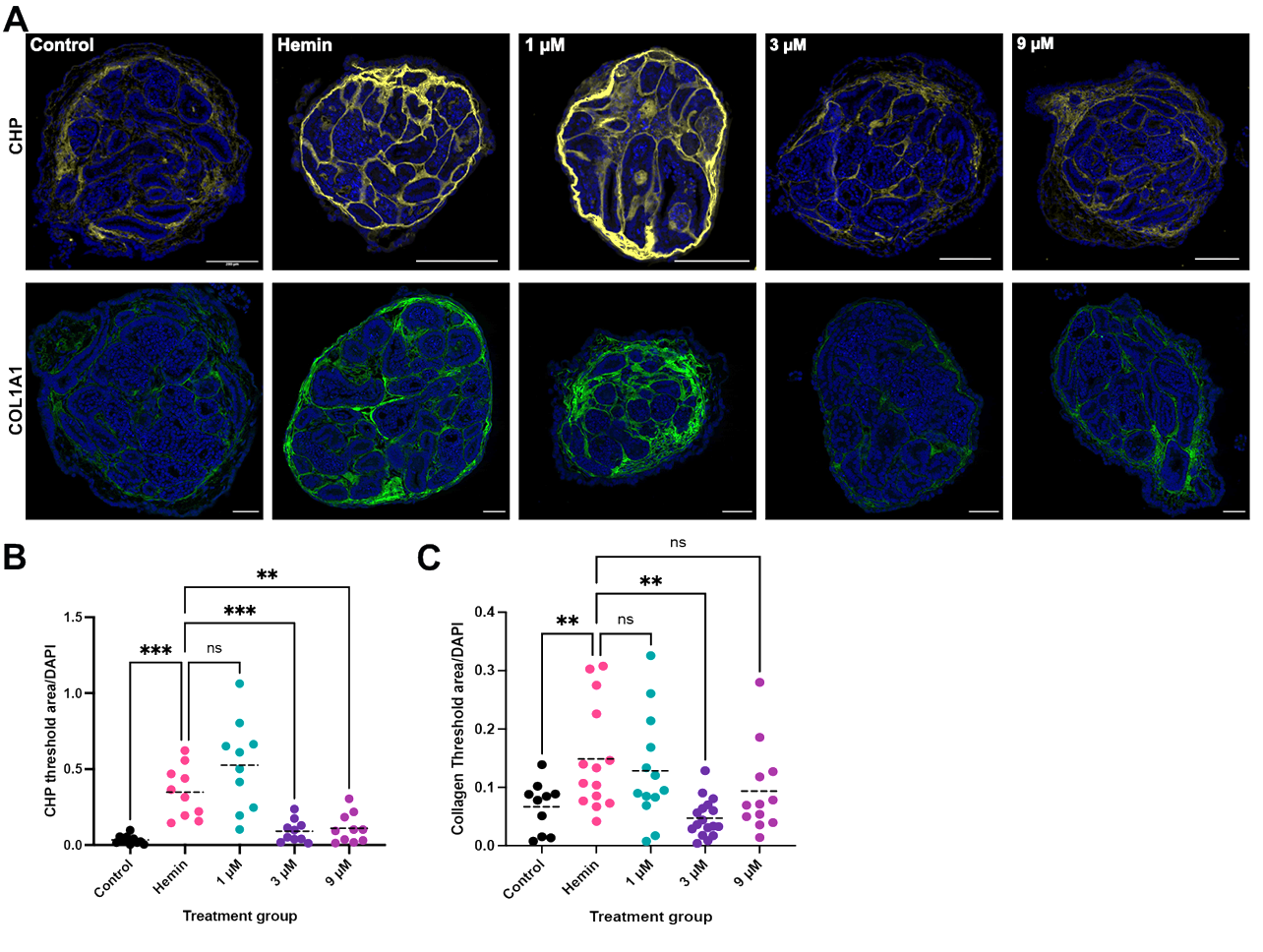


**Supplementary Figure 10. Treatment with UPHD25 reduces collagen induction in kidney organoids.**

**A)** Representative images of paraffin sections of kidney organoids at day 26, stained with collagen hybridizing peptide (CHP; yellow) and collagen 1a1 antibody (COL1A1; green), DAPI staining nuclei (blue). UPHD25 concentrations 1, 3, and 9 µM. **B)** Quantification of CHP staining. Each point represents CHP threshold area/DAPI per organoid, per section. **C)** Quantification of COL1A1 staining. Each point represents COL1A1 threshold area/DAPI per organoid, per section. One-way ANOVA with multiple comparisons to hemin control. ns = non-significant; **P ≤ 0.01; ***P ≤ 0.001. Cell line MANZ4-37. Scale bar = 100 µm.
